# Two-year outcomes of patients presenting to Sydney Eye Hospital with neovascular glaucoma

**DOI:** 10.1007/s10792-023-02675-5

**Published:** 2023-03-13

**Authors:** Ezekiel J. Kingston, Jed A. Lusthaus

**Affiliations:** 1 Department of Ophthalmology, Sydney Eye Hospital, 8 Macquarie Street, Sydney, 2000 Australia; 2 Present Address: Discipline of Ophthalmology, The University of Sydney, Camperdown, Australia

**Keywords:** Glaucoma, Neovascular, Neovascular glaucoma, Retinal ischemia

## Abstract

**Background:**

Neovascular glaucoma (NVG) is a sight-threatening condition that is often refractory to treatment. Current management principles are yet to be standardized due to lack of evidence. We studied the interventions used to treat NVG at Sydney Eye Hospital (SEH) and the two-year surgical outcomes.

**Methods:**

We performed a retrospective audit of 67 eyes of 58 patients with NVG from January 1, 2013, to December 31, 2018. Intraocular pressure (IOP), best-corrected visual acuity (BCVA), number of medications, repeat surgery, recurrent neovascularization, loss of light perception and pain were studied.

**Results:**

The average age of the cohort was 59.67 years (SD 14.22). The most common etiologies were proliferative diabetic retinopathy (35 eyes; 52.2%), central retinal vein occlusion (18 eyes; 26.9%) and ocular ischemic syndrome (7 eyes; 10.4%). 70.1% of eyes (47) received vascular endothelial growth factor injections (VEGFI), 41.8% (28 eyes) received pan-retinal photocoagulation (PRP) and 37.3% (25 eyes) received both prior to or within the first week of presentation to SEH. The most common initial surgical interventions were trans-scleral cyclophotocoagulation (TSCPC) (36 eyes; 53.7%) and Baerveldt tube insertion (18 eyes; 26.9%). 62.7% of eyes (42 eyes) failed (IOP > 21 or < 6 mmHg for two consecutive reviews, further IOP-lowering surgery or loss of light perception) during follow-up. Initial TSCPC failed in 75.0% (27/36 eyes) compared with 44.4% (8/18 eyes) after Baerveldt tube insertion.

**Conclusion:**

Our study reinforces the refractory nature of NVG, often despite intensive treatment and surgery. Improvements in patient outcomes may be achieved with earlier consideration of VEGFI and PRP. This study identifies the limitations of surgical interventions for NVG and highlights the need for a standardized management approach.

## Introduction

Neovascular glaucoma (NVG) is an aggressive secondary glaucoma caused by anterior segment neovascularization leading to synechial angle closure and ocular hypertension. Iris neovascularization was first reported by Bader in 1868 [1]; however, it was not until 1963 that NVG was described by Weiss et al. [2] Further research discovered that NVG is caused by retinal ischemia [3]. NVG is most commonly a secondary complication of proliferative diabetic retinopathy (PDR), central retinal vein occlusion (CRVO) or ocular ischemic syndrome (OIS). [3]

Neovascular glaucoma has poor visual outcomes despite medical and surgical management and at times can require evisceration or enucleation to alleviate pain from a blind eye. Up to 50% of patients with NVG can be left with no light perception if poorly managed [4]. NVG accounts for approximately 3.9–4.3% of all glaucoma [5, 6] and 14% of secondary glaucoma [5] with an incidence of approximately 6.6 people per 100,000 reported in one study [7]. Limited population data exist in the literature. NVG seems to have a higher prevalence in males compared to females although the reason for this is unknown [6, 7].

### Management

Management of NVG is governed by two main treatment principles: reducing the neovascularization drive and controlling IOP [8]. Halting neovascularization can maintain IOP control; however, this may be temporary or inadequate [9].

Suppressing the neovascular drive is achieved by reducing the production of vascular endothelial growth factor using vascular endothelial growth factor inhibitors (VEGFI) (most commonly bevacizumab, although ranibizumab and aflibercept have also been used), pan-retinal photocoagulation (PRP) and peripheral retinal cryotherapy. Management of the underlying systemic condition is also critical as this can reduce the likelihood of recurrent neovascularization. Poor glycemic control is associated with development and recurrence of NVG in patients with proliferative diabetic retinopathy [7].

Ocular hypertension in NVG is managed in a similar stepwise approach to any other type of glaucoma, but treatment escalation is often more urgent. Initially medical therapies are implemented, although surgery is often required. The most common surgical interventions include trans-scleral cyclodiode photocoagulation (TSCPC), glaucoma drainage devices (GDD) and trabeculectomy with mitomycin C (MMC).

## Methods

We performed a retrospective audit of patients presenting to SEH with NVG between January 1, 2013, and December 31, 2018. Inclusion and exclusion criteria are given in Table 1. NVG cases were selected using ‘PC067: SNOMED Diagnosis by Enc,’ a search engine found within Cerner electronic medical records (eMR), as well as ‘the international statistical classification of disease and related health problems, tenth revision, Australian Modification’ (ICD 10-AM). Both databases were searched because SEH uses paper-based notes for outpatients and an eMR for inpatients.

**Table 1:** Inclusion and exclusion criteria

Inclusion criteria	Exclusion criteria
Diagnosis of NVG or rubeotic glaucoma by ophthalmologist that has been documented	Initially reviewed for management of NVG or rubeotic glaucoma at SEH prior to January 1, 2013, or after December 31, 2018
OR Reviewed at SEH for management of neovascular glaucoma	No documentation of raised IOP or signs of neovascularization of iris or angle

*NVG* neovascular glaucoma, *SEH* Sydney Eye Hospital

A total of 93 patients were identified using inclusion and exclusion criteria. Of the 93 patients, five were excluded due to loss of follow-up and a further three were excluded because the diagnosis of NVG was not adequately documented. A further 27 patients were excluded from data analysis due to less than six months of documented follow-up, leaving 67 eyes from 58 patients remaining in the study. Data was collected from hospital medical records and private practices if applicable.

The initial presentation was defined as the patient’s first presentation to SEH; however, if there was no documentation of initial review at SEH prior to surgery, then the referring ophthalmologist’s review was used. The records were then searched to determine whether the patient required surgical intervention, which was then used as the start of the 24-month follow-up period. If surgery was not required within the first year after initial presentation, then the surgery they required was recorded as nil and the starting point was the initial presentation. Further details such as preoperative VEGFI and PRP, etiology and number of IOP-lowering medications were recorded. Patients who received either PRP or had a VEGFI injection intraoperatively within the first week of initial presentation were grouped with the patients who had these interventions preoperatively for data analysis purposes.

Data were collected at one month, 6 months, 12 months, 18 months and 24 months postoperatively. If patients were not reviewed within 3 months of the defined time, no data were recorded for that review. If patients did not have documentation regarding neovascularization, complications or pain during the review, then it was recorded as unknown.

### Statistical analysis

Data analysis was completed using SPSS software version 27 (SPSS, Inc., Armonk, NY). Study population details were analyzed to find the number and percentage of the cohort that had each of the string-based variables. Numerical data were then analyzed to determine whether it was parametric or nonparametric using Kolmogorov–Smirnov test. Means and medians were recorded with standard deviations (SD) and interquartile ranges (IR) within each set of numerical data. Normally distributed data had means recorded with standard deviations, whereas nonparametric data had medians recorded with interquartile ranges. All variables were found to be nonparametric other than preoperative IOP. Related-samples Wilcoxon signed rank test and Mann–Whitney U and Kruskal–Wallis tests were used to determine statistically significant differences between variables.

Kaplan–Meier survival analysis was completed to analyze time to failure for the whole cohort as well as time to failure for each subsection of the cohort based on initial surgical intervention. Failure was defined as IOP > 21 or < 6 mmHg for two consecutive postoperative reviews, requiring further IOP-lowering operations or loss of light perception. Chi-squared test and log-rank (Mantel–Cox) tests were then used to determine whether there was any statistically significant difference in survival duration between each of the surgical intervention group. A two-sided p-value less than 0.05 was considered statistically significant.

## Results

Patient demographics are given in Table 2. Median follow-up time was 24 months (IR 6), and the mean was 20.42 months (SD 6.01, range 6 months to 6 years). The most common causes of NVG within the cohort were PDR (35 eyes; 52.2%) followed by CRVO (18 eyes; 26.9%) and OIS (7 patients 10.4%**)**. BRVO (3 eyes), chronic retinal detachment (2 eyes), CRAO (1 eye) and Sticklers syndrome (1 eye) were found to be less common.

**Table 2:** Baseline characteristics

Demographic	*N* = 67
Mean age	59.67 years (SD 14.22; range 26–89 years; parametric data p = 2)
Gender	Males: 37 eyes (55.2%) of 30 people Females: 30 eyes (44.8%) of 28 people
Comorbidities	Diabetes mellitus 53 eyes (79.1%) of 44 people Systemic hypertension 41 eyes (61.2%) of 38 people Diabetes and Systemic hypertension 35 eyes (52.2%) of 31 people
Place of birth	- Australia and not identifying as Aboriginal or Torres Strait Islander: 29 eyes, 26 patients - Australia and identifying as Aboriginal: 1 eye, 1 patient - Europe: 15 eyes, 11 patients - Asia: 10 eyes, 9 patients - Southeast Asia: 4 eyes, 3 patients - South America: 1 eye, 1 patient - Middle east: 3 eyes, 3 patients - Oceania islands: 4 eyes, 4 patients
Lens Status	Documented pseudophakia 23 eyes (34.3%)

### Management of neovascularization

Forty-seven patients (70.1%) received intravitreal VEGFI and 28 patients (41.8%) received PRP within the first week of presentation or prior to presentation for management of NVG (Table 3). Cryotherapy was used in one patient intraoperatively in combination with TSCPC. No statistically significant differences in IOP control were found between those who had intravitreal bevacizumab or PRP before or within the first week of presentation.

**Table 3:** Interventions to treat neovascularization

Intervention	Number of eyes (%)
Intravitreal bevacizumab before or within the first week from presentation	47 (70.1%) BVT 14 (77.8%) TSCPC 24 (66.7%) Nil 5 (62.5%) PPV 3 (100%) Trab + MMC 1 (50%)
Intravitreal bevacizumab before or within the first month from presentation	54 (80.6%) BVT 16 (88.9%) TSCPC 29 (80.6%) Nil 5 (62.5%) PPV 3 (100%) Trab + MMC 1 (50%)
PRP before or within the first week of presentation	28 (41.8%) BVT 8 (44.4%) TSCPC 14 (38.9%) Nil 3 (37.5%) PPV 3 (100%)
PRP before or within the first month from presentation	42 (62.7%) BVT 13 (72.2%) TSCPC 22 (61.1%) Nil 4 (50.0%) PPV 3 (100%)
PRP prior to NVG diagnosis without additional top-up	7 (10.4%)
Postoperative PRP top-up	27 (40%)
Cryotherapy 1 month post-diagnosis	1 (1.5%)

*VEGFI* vascular endothelial growth factor inhibitor, *PRP* pan-retinal photocoagulation, *BVT* Baerveldt tube, *TSCPC* trans-scleral cyclophotocoagulation, *PPV* pars plana vitrectomy, *Trab + MMC* trabeculectomy with mitomycin C

### Management of ocular hypertension

Initial surgical intervention occurred in 88.1% of eyes within the first year of presenting to SEH. All procedures were used to lower IOP except for pars plana vitrectomy (PPV) in three eyes (4.5%), two with anterior chamber washout. TSCPC and BVT were the most common IOP-lowering surgeries, accounting for 36 eyes (53.7%) and 18 eyes (26.9%), respectively. The only other surgical intervention to lower IOP was trabeculectomy with MMC (2 eyes, 3.0%).

### Outcomes of management among the whole cohort

Median BCVA declined over the study period from 1.9 log units (IR 1.7) to 2.30 log units (IR 2.00). Median IOP and number of medications also were seen to decline (Table 4).

**Table 4:** Visual acuity and intraocular pressure outcomes after initial review

	Initial SEH review (*N* = 67)	1 month (*N* = 58)	6 months (*N* = 59)	12 months (*N* = 51)	18 months (*N* = 45)	24 months (*N* = 46)
BCVA (LogMAR)	1.90 (IR 1.7)	1.90 (IR 1.58)	1.90 (IR 2.22)	2.30 (IR 1.82)	2.30 (IR 2.22)	2.30 (IR 2.00)
IOP (mmHg)	39.00 (IR 22)	17.00 (IR 24)	14.00 (IR11)	15.50 (IR 10)	14.00 (IR 12)	16.00 (IR 15)
Medications	4 (IR 2)	3 (IR 3)	3 (IR 4)	2 (IR 4)	1 (IR 3)	1 (IR 3)
No. of missed data points	0 BCVA 0 IOP 0 Meds	10 BCVA 9 IOP 9 Meds	9 BCVA 8 IOP 9 Meds	16 BCVA 16 IOP 17 Meds	24 BCVA 24 IOP 23 Meds	21 BCVA 24 IOP 22 Meds

*BCVA* best-corrected visual acuity, *IOP* intraocular pressure

Related-samples Wilcoxon signed rank test was performed on each variable at every follow-up to compare the median values of IOP, BCVA and number of medications. A significant decrease in median IOP was found when comparing the initial IOP to each of the five follow-up IOPs (*p* < 0.001). There was also a significant decrease in median number of IOP-lowering medications required at each follow-up when compared to initial review (*p* < 0.001). No statistically significant difference in median BCVA was found between initial BCVA when compared to BCVA at each follow-up period (*p* > 0.05), except for at 12 months where there was a significant decrease (*p* < 0.001). BCVA worsened at 12 months, possibly due to retinal ischemia or glaucoma. Also patients with better visual outcomes may have returned to their referring ophthalmologist for ongoing review. At one-month follow-up, 40.4% (*N* = 23) of eyes had improved BCVA, 36.8% (*N* = 21) had worse BCVA and 22.8% (*N* = 13) had no change in BCVA from initial review. This worsened to 15.2% (7 eyes), 65.2% (30 eyes) and 19.6% (9 eyes), respectively, after 24 months.

The most common complications of NVG and its management were hypotony (a total of 11 eyes: three at one month, two at six months, three at 12 months, one at 18 months and a further two at 24 months), hyphema (five eyes at six months and one at 12 months) and corneal edema (four eyes at six months and two at 24 months). Of the 11 hypotonous eyes, six initially had a BVT, of which three had further IOP-lowering surgeries prior to becoming hypotonous. Four of the eyes initially had TSCPC, although all four of these had further IOP-lowering surgeries prior. Only one had no surgery but prior to becoming hypotonous had TSCPC. Blebitis occurred in one eye that had initially undergone Trabeculectomy with MMC. Table 5 outlines occurrences of pain, anterior segment neovascularization or further surgical intervention at each follow-up. All percentages are taken as valid percentages of the cohort at each review.

**Table 5:** Indicators of treatment failure during postoperative follow-up

	1 month (*N* = 58)	6 months (*N* = 59)	12 months (*N* = 51)	18 months (*N* = 45)	24 months (*N* = 46)
Pain	9 eyes (15.5%)	6 eyes (10%)	3 (5.9%)	1 (2.2%)	4 (8.7%)
Neovascularization of anterior segment	10 eyes (17.2%)	11 eyes (18.3%)	1 (2%)	1 (2.2%)	3 (6.5%)
Further surgical intervention	7 eyes (12.1%)	12 eyes (20.3%)	8 eyes (15.4%)	5 eyes (13%)	5 eyes (10.9%)
Hyphaema	2 eyes (3.4%)	5 eyes (8.3%)	1 eye (2.0%)	0 eyes	0 eyes
Hypotony	3 (5.2%)	3 (5.1%)	4 (7.8%)	4 (8.9%)	4 (8.7%)
Loss of light perception (cumulative)	6 eyes	11 eyes	16 eyes	16 eyes	20 eyes
Additional surgery required	3 TSCPC 2 BVT 1 BVT revision 1 TSCPC + BVT	3 BVT 8 TSCPC 1 BVT removal	6 TSCPC 1 TSCPC + PPV 1 Enucleation	1 TRAB 1 Gunderson flap 1 TSCPC 1 BVT 1 BVT revision	2 TSCPC 1 BVT removal 1 TRAB 1 Enucleation

*TSCPC* trans-scleral cyclophotocoagulation, *BVT* Baerveldt tube, *PPV* pars plana vitrectomy, *TRAB* trabeculectomy and mitomycin C, *NVI* neovascularization of the iris

### Reviewing outcomes of management based on initial surgical intervention

#### Potential confounders

Two eyes in the TSCPC group had BCVA of 3.0 and seven had BCVA of 2.7, whereas in the BVT group no eyes had BCVA of 3.0 and only one had BCVA of 2.7. Hence, more eyes within the TSCPC group were managed with conservative goals of management.

Age and number of comorbidities were analyzed. There was a significant difference in median age between the trabeculectomy with MMC group (28.5 years) when compared to BVT (58 years) and TSCPC (63 years) (Mann–Whitney *p* < 0.05, *Z* = −2.3 and *p* < 0.05, *Z* = −2.4 respectively). No other significant differences were found in median ages between the groups.

There were no statistical differences in median number of comorbidities between each initial surgical intervention group (Mann–Whitney *p* > 0.05). Median times to intervention for each group were also similar (BVT 26 days (IR 108), TSCPC 15 days (IR 73)). No significant difference in follow-up duration was found between each intervention group (Mann–Whitney test *p* > 0.05).

#### Median IOP between each intervention group

Median IOP in each surgical group was calculated at the initial and follow-up reviews. Significant differences in IOP occurred between some groups during follow-up and are reported in Table 6. The numbers of patients who had IOP within normal limits at 24 months were similar in each group (70% in BVT, 61% in TSCPC, 50% in PPV). Both patients who had initial trabeculectomy and MMC had IOP within normal limits (≥ 6 mmHg and ≤ 21 mmHg) for all reviews (up to 12 and 18 months).

**Table 6:** Median IOP with interquartile ranges at each follow-up interval

Surgery	Initial review IOP (mmHg) (*N* = 67)	1-month IOP (mmHg) (*N* = 57)	6-month IOP (mmHg) (*N* = 59)	12-month IOP (mmHg) (*N* = 51)	18-month IOP (mmHg) (*N* = 44)	24-month IOP (mmHg) (*N* = 43)
BVT (N = 18)	38.5 (IR 25)	14.0 (IR 17)	14.0 (IR 10)	14.0 (IR 7)	11.0 (IR 5)	13.0 (IR 17)
TSCPC (*N* = 36)	40.5 (IR 21)	22 (IR 28)	17.0 (IR 19)	18.5 (IR 11)	17.0 (IR 13)	19.0 (IR 11)
Delayed or no surgery (*N* = 8)	31.0 (IR 13)	17.5 (IR 18)	16.0 (IR 26)	23.0 (IR 32)	22.0 (IR 15)	14.5 (IR 18)
PPV (*N* = 3)	29.0 (IR nil)	15.0 (IR nil)	11.0 (IR nil)	32.0 (IR nil)	40.0 (IR nil)	37.0 (IR nil)
Trab + MMC (*N* = 2)	36.0 (IR nil)	8.0 (IR nil)	8.0 (IR 0)	14.5 (IR nil)	Nil	Nil
Statistically significant difference Mann–Whitney test (for two variables) and Kruskal–Wallis for all variables	1. BVT vs TSCPC vs Delayed or no surgery vs PPV vs Trab + MMC (*p* = 0.124)	1. BVT vs TSCPC (*p* < 0.028, *Z* = −2.2) 2. BVT vs TSCPC vs Delayed or no surgery vs PPV vs Trab + MMC (*p* = 0.069)	1. BVT vs TSCPC vs Delayed or no surgery vs PPV vs Trab + MMC (*p* = 0.127)	1. BVT vs TSCPC (*p* < 0.022, *Z* = −2.3) 2. BVT vs TSCPC vs Delayed or no surgery vs PPV vs Trab + MMC (*p* = 0.113)	1. BVT vs TSCPC (*p* < 0.049, *Z* = −2.0) 2. BVT vs TSCPC vs Delayed or no surgery vs PPV vs Trab + MMC (*p* = 0.133)	2. BVT vs TSCPC vs Delayed or no surgery vs PPV vs Trab + MMC (*p* = 0.798)

*BVT* Baerveldt tube, *TSCPC* trans-scleral cyclophotocoagulation, *PPV* pars plana vitrectomy, *Trab + MMC* trabeculectomy and mitomycin C

#### BCVA in each surgical group

Median BCVA was calculated for each surgical group at the initial review and each successive follow-up. The TSCPC group had a worse median BCVA than the BVT group throughout follow-up although this was only found to be statistically significant at 1- and 6-month reviews (Mann–Whitney *p* < 0.05, *Z* < −2.0) (Table 7). This lack of significant results is likely due to a higher attrition rate as time progressed.

**Table 7:** Median BCVA with interquartile ranges at each follow-up interval

Surgery	Initial r/v BCVA (LogMAR) (*N* = 67)	1-month BCVA (LogMAR) (*N* = 56)	6-month BCVA (LogMAR) (*N* = 58)	12-month BCVA (LogMAR) (*N* = 51)	18-month BCVA (LogMAR) (*N* = 44)	24-month BCVA (LogMAR) (*N* = 46)
BVT (*N* = 18)	0.94 (IR 1.51)	0.94 (IR 1.27)	0.90 (IR 1.82)	1.39 (IR 1.40)	1.90 (IR 1.70)	1.90 (IR 2.10)
TSCPC (*N* = 36)	2.30 (IR 1.53)	2.30 (IR 1.52)	2.30 (IR 1.34)	2.30 (IR 1.70)	2.30 (IR 1.85)	2.30 (IR 1.46)
Delayed or no surgery (*N* = 8)	0.80 (IR 2.17)	1.15 (IR 1.66)	0.44 (IR 1.27)	1.80 (IR 2.73)	1.48 (IR 2.82)	1.09 (IR 2.82)
PPV (*N* = 3)	2.70 (IR 0)	1.90 (IR nil)	1.90 (IR nil)	2.45 (IR nil)	2.45 (IR nil)	2.45 (IR nil)
Trab + MMC (*N* = 2)	1.34 (IR nil)	0.45 (IR nil)	0.39 (IR nil)	0.74 (IR nil)	Nil	Nil
Statistically significant difference Mann–Whitney test (for two variables) and Kruskal–Wallis for all variables	1. TSCPC vs BVT (*p* = 0.017, *Z* = −2.4) 2. BVT vs TSCPC vs Delayed or no surgery vs PPV vs Trab + MMC (*p* = 0.021)	1. TSCPC vs BVT (*p* = 0.005, *Z* = −2.8) 2. BVT vs TSCPC vs Delayed or no surgery vs PPV vs Trab + MMC (*p* = 0.017)	1. TSCPC vs BVT (*p* < 0.008, *Z* = −2.7) 2. 1. BVT vs TSCPC vs Delayed or no surgery vs PPV vs Trab + MMC (*p* = 0.011)	1. BVT vs TSCPC vs Delayed or no surgery vs PPV vs Trab + MMC (*p* = 0.147)	1. BVT vs TSCPC vs Delayed or no surgery vs PPV vs Trab + MMC (*p* = 0.828)	1. BVT vs TSCPC vs Delayed or no surgery vs PPV vs Trab + MMC (*p* = 0.521)

*BVT* Baerveldt tube, *TSCPC* trans-scleral cyclophotocoagulation, *PPV* pars plana vitrectomy, *Trab + MMC* trabeculectomy and mitomycin C

#### Number of IOP-lowering medications in each surgical group

The final variable that was used to determine outcomes of each surgical intervention was the number of IOP-lowering medications that were being used at the initial and then subsequent follow-up reviews (Table 8). There were no statistically significant differences between each surgical group using Mann–Whitney test.

**Table 8:** Median number of IOP-lowering medications with interquartile ranges at each review

Surgery	Initial *r*/*v* (*N* = 66)	1 month (*N* = 57)	6 months (*N* = 58)	12 months (*N* = 50)	18 months (*N* = 45)	24 months (*N* = 45)
BVT (*N* = 18)	5.0 (IR 1)	3.0 (IR 4)	3.0 (IR 4)	2.0 (IR 3)	1.5 (IR 2)	1.0 (IR 3)
TSCPC (*N* = 36)	4.0 (IR 1)	4.0 (IR 2)	3.0 (IR 3)	3.0 (IR 3)	1.0 (IR 3)	1.0 (IR 3)
Delayed or no surgery (*N* = 8)	3.0 (IR 2)	1.5 (IR 3)	1.0 (IR 2)	0.0 (IR 4)	0.0 (IR 2)	0.0 (IR 2)
PPV (*N* = 3)	3.0 (IR nil)	3.0 (IR nil)	1.0 (IR nil)	2.5 (IR nil)	2.0 (IR nil)	2.0 (IR nil)
Trab + MMC (*N* = 2)	4.5 (IR nil)	0.0 (IR nil)	Nil	Nil	Nil	Nil
Statistically significant difference Mann–Whitney test (for two variables) and Kruskal–Wallis for all variables	1. BVT vs Delayed or no surgery *p* = 0.011, *Z* = −2.6 2. BVT vs TSCPC vs Delayed or no surgery vs PPV vs Trab + MMC (*p* = 0.018)	1. BVT vs TSCPC vs Delayed or no surgery vs PPV vs Trab + MMC (*p* = 0.079)	1. BVT vs TSCPC vs Delayed or no surgery vs PPV vs Trab + MMC (*p* = 0.219)	1. BVT vs TSCPC vs Delayed or no surgery vs PPV vs Trab + MMC (*p* = 0.398)	1. BVT vs TSCPC vs Delayed or no surgery vs PPV vs Trab + MMC (*p* = 0.328)	1. BVT vs TSCPC vs Delayed or no surgery vs PPV vs Trab + MMC (*p* = 0.521)

*BVT* Baerveldt tube, *TSCPC* trans-scleral cyclophotocoagulation, *PPV* pars plana vitrectomy, *Trab + MMC* trabeculectomy and mitomycin C

#### Failure

Each review was used to determine whether a patient’s initial surgical intervention had failed. The review time at which failure occurred was recorded and then compared between three different surgical groups: BVT, TSCPC and other (Table 9). The overall survival analysis was conducted using the Kaplan–Meier survival analysis and log-rank (Mantel–Cox) Chi-squared analysis.

**Table 9:** Failure in each initial surgical intervention group where failure is defined as IOP > 21 or < 6 mmHg for two consecutive reviews or where further IOP-lowering operation was required or where loss of light perception occurred

Intervention	Total number of eyes	Number of eyes that failed during follow-up	Percentage of eyes that failed during follow-up	Mean follow-up duration
BVT	18	8	44.4%	20.3 (SD 5.5)
TSCPC	36	27	75.0%	20.3 (SD 6.4)
Other	13	7	53.8%	21.0 (SD 6.0)
Overall	67	42	62.7%	20.4 (SD 6.0)

*BVT* Baerveldt tube, *TSCPC* trans-scleral cyclophotocoagulation, *Other* delayed or no surgery, pars plana vitrectomy and trabeculectomy with mitomycin C groups

Eight NVG patients did not require surgery within the first year and were followed for two years from their presentation to SEH. They cannot be compared with surgical patients who were followed for two years from their initial operation. Of the patients who did not require surgery, two ended up having surgery (PPV and TSCPC) more than one year after initial presentation. Of those who did not initially require surgery, 62.5% had VEGFI and 37.5% had PRP prior to or within the first week of presenting. All patients who underwent PPV received both VEGFI and PRP within the first week. An increased rate of VEGFI and PRP use within the first week of presentation, when compared to the TSCPC group, likely contributed to the reasonable outcomes seen in these groups.

**Fig. 1:**
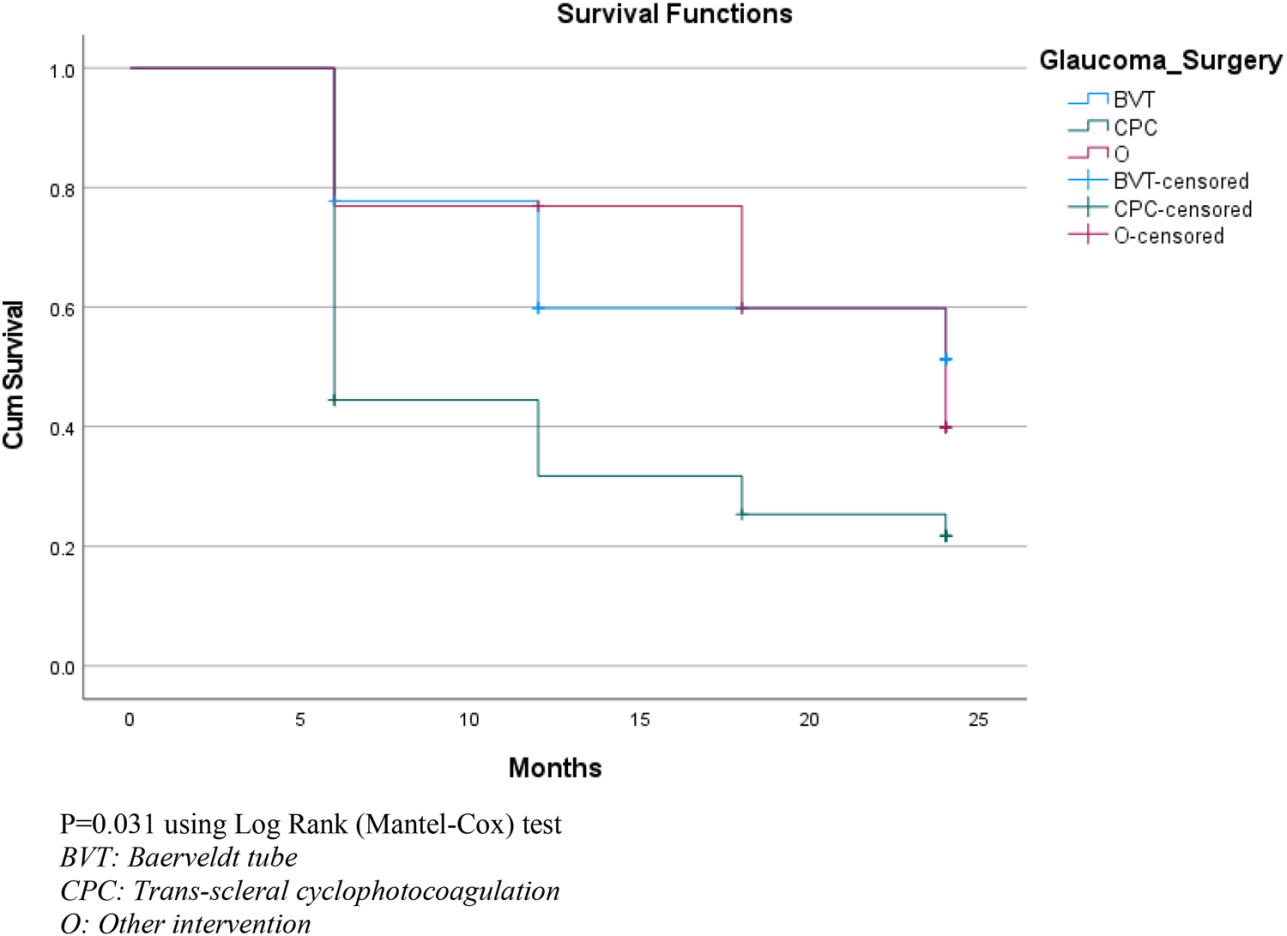
Kaplan–Meier survival curve of surgical intervention. *P* = 0.031 using log-rank (Mantel–Cox) test. BVT: Baerveldt tube. CPC: Trans-scleral cyclophotocoagulation. O: Other intervention

Kaplan–Meier survival analysis demonstrated surgical failure in 62.7% of eyes with an estimated median survival time of 12 months (95% median CI 4.109–19.891). The estimated median survival time was 24 months for BVT and 6 months for TSCPC; however, no 95% median confidence interval (CI) could be determined using the data that were available. This is demonstrated in Fig. 1 where the cumulative survival drops below 0.5. Each step in the graph represents the number of cases failing at each follow-up period. There were significantly better estimated median survival times seen in eyes which were managed with BVT when compared to those who were managed with TSCPC when using log-rank (Mantel–Cox) Chi-squared test to complete pairwise analysis (*p* = 0.022). There were no significant differences when comparing both TSCPC and BVT with the other group.

Further surgical intervention occurred in 30 eyes. Interventions included 19 TSCPC’s, 7 BVT insertion’s, 2 BVT removals (both in the same eye due to hypotony), 3 BVT revisions, 2 trabeculectomies with MMC, 2 enucleation and 1 Gunderson flap. Twenty-eight eyes failed due to IOP being outside the normal range of 6–21 mmHg for two consecutive follow-ups and 20 eyes failed due to loss of light perception. It is important to note that the causes for failure were multifactorial for many of the eyes and therefore, fit into multiple categories.

Further surgical interventions were more common in eyes which initially received TSCPC (21 eyes; 58.3%) when compared to all other intervention groups. BVT group had 5 eyes (27.8%), PPV had 1 (33%), delayed or no surgery 2 (25%) and trabeculectomy with MMC 0 eyes.

To account for the potential confounding caused by SEH’s protocol stating that those patients with BCVA of 3.0 log units should be managed with TSCPC to keep the eye comfortable, we excluded these patients. This demonstrated slight improved outcomes for all groups and hence the protocol was not a significant confounder (failure rates of 70.4% in TSCPC group, 41.2% in the BVT group and 57.7% overall). When also excluding those with BCVA of 2.7 at initial review, failure rates were 69.2% in TSCPC group, 37.5% in BVT group and 57.7% overall. There was also still a statistically significant difference between the TSCPC and BVT groups using log-rank (Mantel–Cox) test (*p* = 0.035).

## Discussion

Neovascular glaucoma is a severe sight-threatening condition that has a very poor long-term visual prognosis. We have described 2-year outcomes of NVG patients presenting to a quaternary referral center. The results of two commonly used surgical interventions for the treatment of NVG, namely TSCPC and BVT, were compared.

The patient cohort identified in this study is similar to other retrospective audits such as those conducted at Songklanagarind Hospital (SH) in Thailand [10], Wenzhou Medical University Hospital (WMUH) in China [11] and the associacion para Evitar la Ceguera in Mexico [12]. The etiology of NVG among this cohort is consistent with the three most common causes of NVG: proliferative diabetic retinopathy, ischemic CRVO and ocular ischemic syndrome [6, 10]. This highlights that NVG may be preventable if underlying risk factors such as diabetes and hypertension are appropriately managed.

Tight control of blood glucose levels has been demonstrated to reduce the likelihood of patients with proliferative diabetic retinopathy developing NVG [13]. High HbA1c levels have been found to be a significant predictor of NVG in patients who have had a PPV for management of PDR [13]. During this audit, it was noted that documentation regarding strict advice to follow-up with an endocrinologist to improve their diabetic control was lacking within the notes. Referral to an endocrinologist and strict counseling about the importance of adherence to diabetic regimens as well as lifestyle changes need to be addressed and documented upon each review.

IOP, BCVA, pain and failure rates at SEH were similar to reports from other eye hospitals and highlight possible areas for improvement in management of NVG. Although angle status was reviewed in these patients, it was not reported due to lack of clear documentation. A significant decrease was seen in both median IOP and median number of IOP-lowering medications from initial review when compared to each follow-up review (*p* < 0.001). However, the number of patients at each follow-up with an IOP between 6 and 21 mmHg was always less than 72% and as low as 56.9% of patients. These figures reflect modest IOP control with the currently available treatments and surgeries in Australia.

Median BCVA within the cohort deteriorated over the 24-month follow-up period, although NVG was only one of the contributing factors to poor visual outcomes. Initial BCVA was 1.9 log units (IR 1.7) and worsened to 2.3 log units (IR 1.82) at 12 months. A similar pattern was seen at SH (mean BCVA at baseline of 1.77 ± 0.76 and final follow-up BCVA of 1.99 ± 0.86) [10]. BCVA worsened in 65.2% of patients at SEH after 24 months of follow-up when compared with baseline. More favorable results were reported at King Khaled Eye specialist hospital (KKEH) in Saudi Arabia (38% of eyes at 24 months) [7, 14] and SH (45% of eyes at 21 ± 18 months) [10]. Non-glaucomatous disease processes that initially lead to NVG may be a confounder when using BCVA as an outcome. PDR, CRVO and OIS can often lead to macular edema or ischemia, both of which can significantly reduce BCVA.

Failure rates in our study highlight the challenges associated with surgical management of NVG. The primary surgery performed on 62.7% of patients within the cohort had failed by the 24-month review. Lower failure rates were seen at SH (IOP > 21 or < 6, further operations, loss of light perception or any severe complications) and WMUH (IOP > 21 or < 6 or any decrease in visual acuity) with similar definitions of failure [10, 11]. SH had a surgical failure rate of 50.3% after an average of 21-month follow-up [10] and WMUH had a 37.4% failure rate at 2 years [10]. The lowest failure rate within SH was seen in the trabeculectomy with MMC and intraocular (intravitreal or intracameral) bevacizumab with a failure rate of 44% [10]. A similar failure rate was seen in the BVT group at SEH (44.4%).

Timely administration of intravitreal VEGFI and application of PRP may improve outcomes for patients with NVG [15–23]. The goal should be for all patients to receive VEGFI on initial presentation and PRP as soon as possible (fundus view permitting). Bevacizumab is the VEGFI of choice for NVG at SEH, which is consistent with reported VEGFI use at other institutions. [15–20] VEGFI use for NVG is ‘off label’ in Australia, and so, the most cost-effective VEGFI choice is bevacizumab. Within this study, 80.6% of patients received VEGFI therapy and 62.7% of patients received PRP (either top-up or initial PRP) prior to or within one month of presentation. This decreased to 70.1% and 41.8%, respectively, when only including those patients who received these interventions prior to, or within, one week of presentation. This highlights that large proportion of patients did not receive these interventions in a timely manner. It was not clearly documented as to why some patients did not receive VEGFI. One patient had a traumatic AC paracentesis in preparation for VEGFI, and hence, VEFI was not inserted. Some patients may have had VEGFI without it being documented. Patients presenting with a dense vitreous hemorrhage or large hyphema were unable to have initial PRP. This was the case for three patients who received intraoperative PRP with PPV ± anterior chamber washout within the first week and hence were included. 80.6% of patients received initial or additional PRP during the study period. An additional 6% of patients had evidence of previous PRP but did not receive additional PRP since diagnosis of NVG. Retinal cryotherapy was rarely used at SEH with only two (3%) patients receiving this. Cryotherapy may warrant further research as favorable outcomes have been demonstrated in a small study combining 360-degree retinal cryotherapy with PRP and IVB in patients with NVG [14, 24].

The timing and choice of IOP-lowering surgical intervention is critical when managing NVG. Choice of surgical intervention in this study was made by each individual glaucoma surgeon and was based on factors such as visual acuity, intraocular pressure and angle status. This has limited the conclusions that can be drawn from this study. The two most selected surgical interventions to lower IOP in patients with NVG at SEH were TSCPC (53.7%) and BVT (26.8%). Other retrospective reviews at KKEH and WMUH demonstrated similar preferences for TSCPC and GDD [7, 11, 14]. IOP was significantly reduced in our study after 24 months regardless of surgery used.

There were no standardized protocols for administration of TSCPC or insertion of BVT at SEH. BVTs were placed both in the AC and in the sulcus depending on surgeon preference. Number and power of shots of TSCPC varied greatly but were titrated to avoid ‘pops.’

This study highlights differences in outcomes between TSCPC and BVT. There was no statistically significant difference in number of systemic comorbidities, age or median BCVA on presentation between those who initially had TSCPC, BVT or another procedure. Median IOP up to 12 months was significantly lower in the BVT group when compared to TSCPC. BVT failure rate during the study was also lower (44.4%) when compared to TSCPC (75.0%). Excluding patients presenting with BCVA of 2.7 log units or 3.0 log units marginally reduced failure rates for BVT and TSCPC were seen.

Further surgical interventions were more common in the TSCPC group when compared to other groups. This is not surprising because TSCPC is difficult to titrate and may be used as a temporizing measure until a more definitive surgery can be performed.

From this audit, we would recommend timely use of VEGFI and PRP with VEGFI to be given at initial presentation if possible. We would also recommend early referral to a general practitioner and/or endocrinologist to manage risk factors for NVG. Finally, we recommend that further research be completed to establish what the most effective surgical intervention is for managing ocular hypertension in NVG.

Neovascular glaucoma is a potentially devastating condition with ocular and systemic implications. Timely intervention is critical, but there is a lack of management standardization particularly at initial presentation. We have outlined retrospective real-world data from SEH, which reflects experiences reported by other international eye centers. Surgical failure rates are high, and prospective studies are required to guide surgical decision making. Immediate consideration of VEGFI and PRP on initial presentation of NVG may improve patient outcomes and reduce the need for surgical interventions.

## Data Availability

Dataset gathered during this study is not available publically due to privacy requirements although it could be completely de-identified and provided in part if requested.
